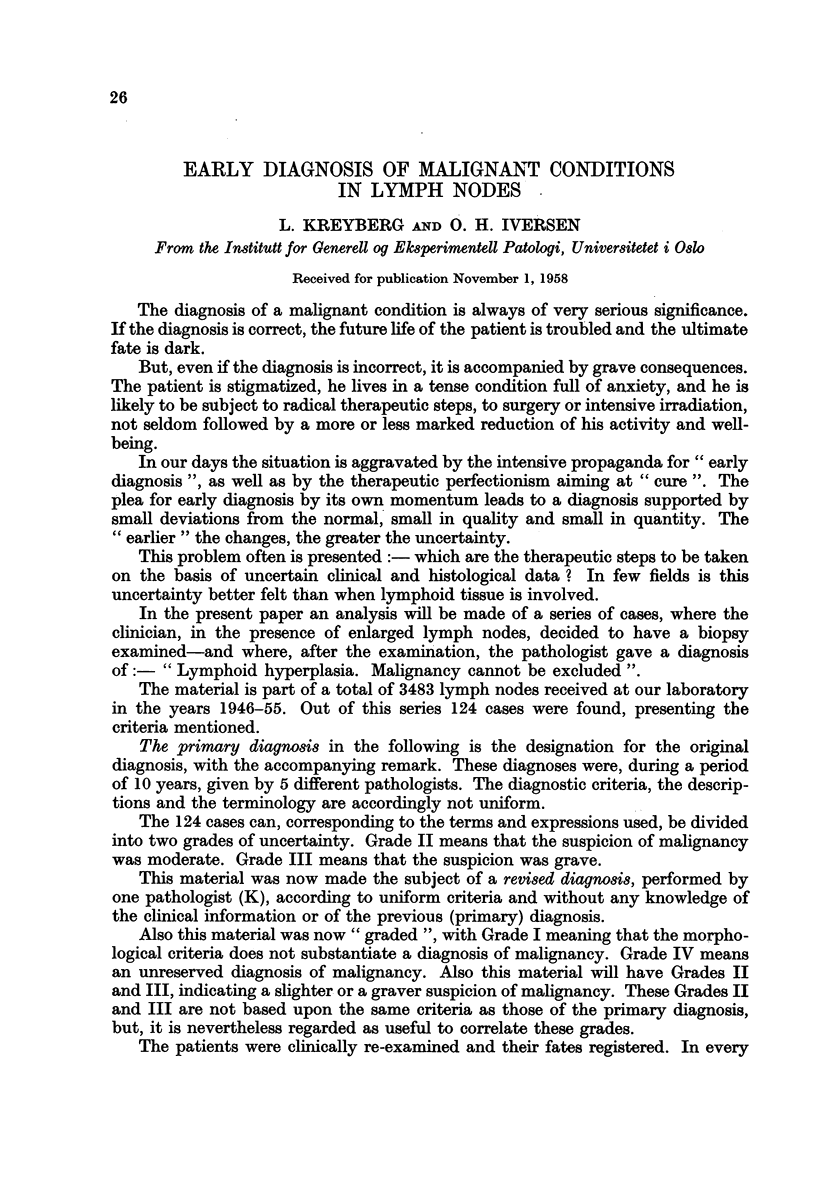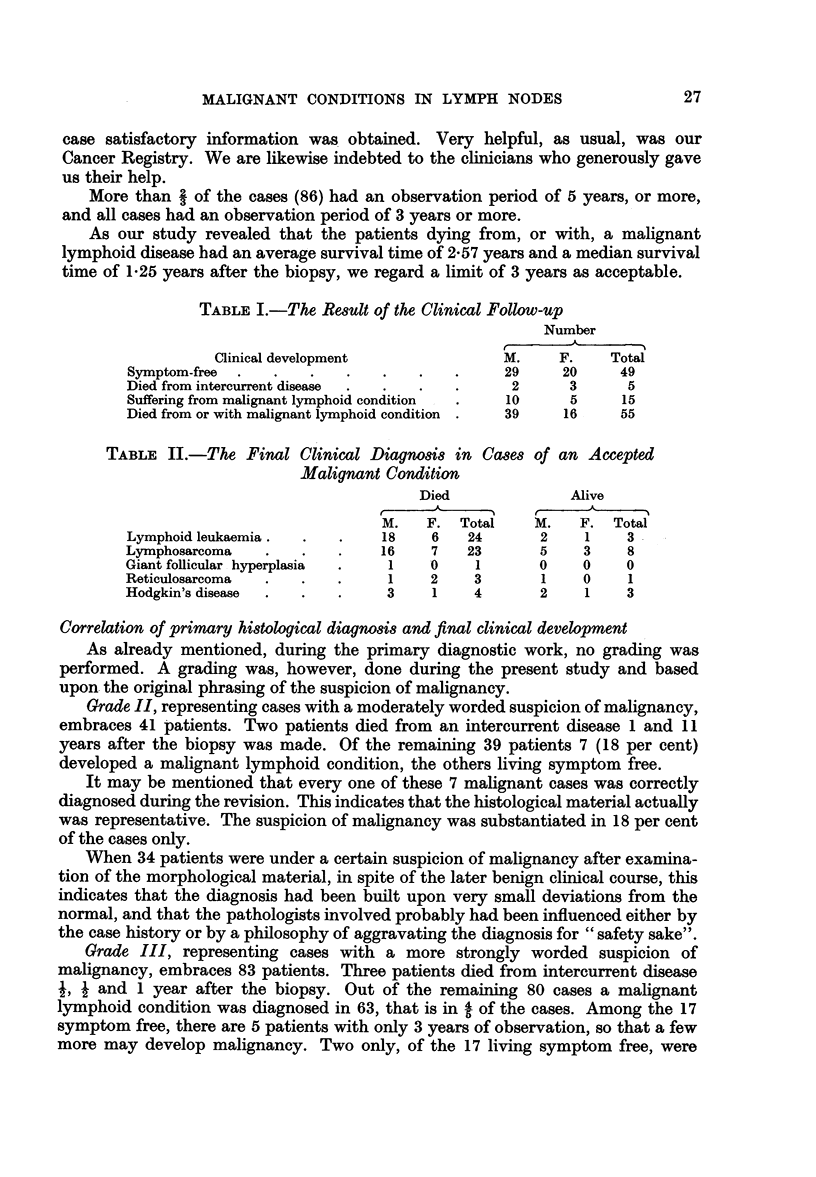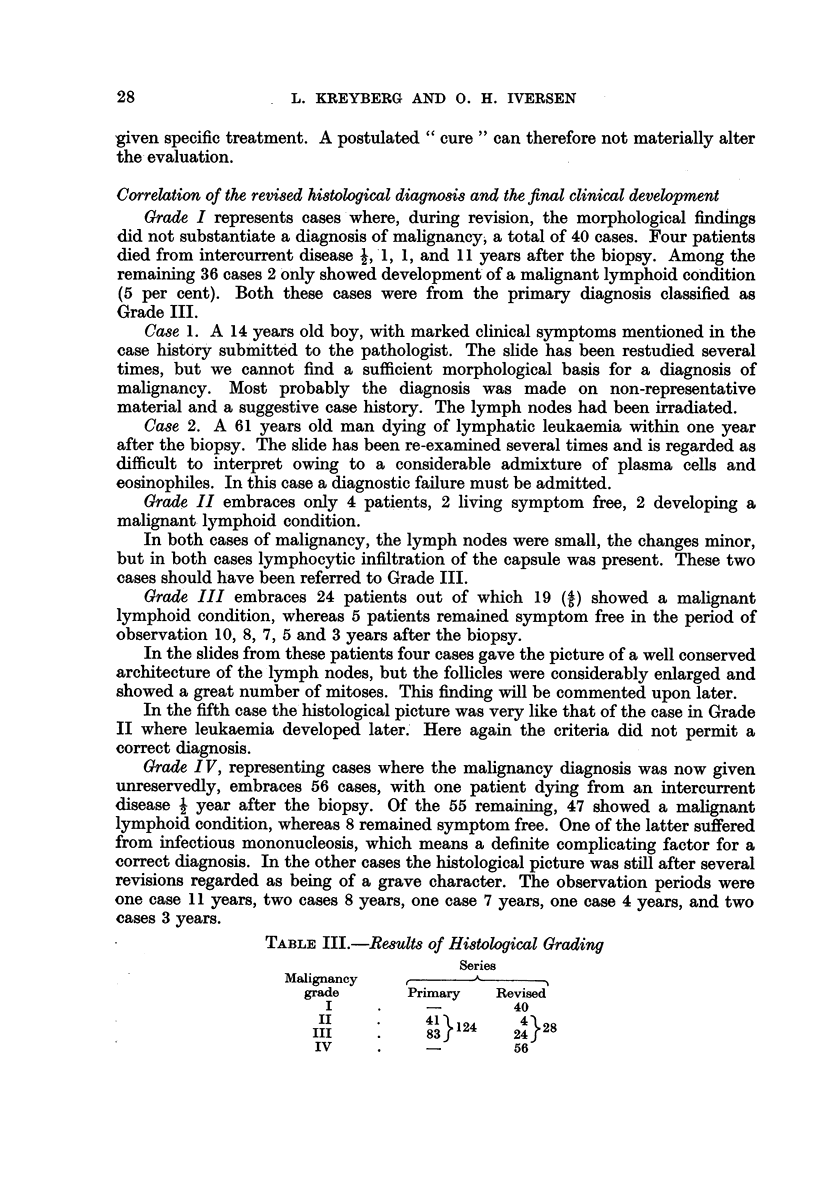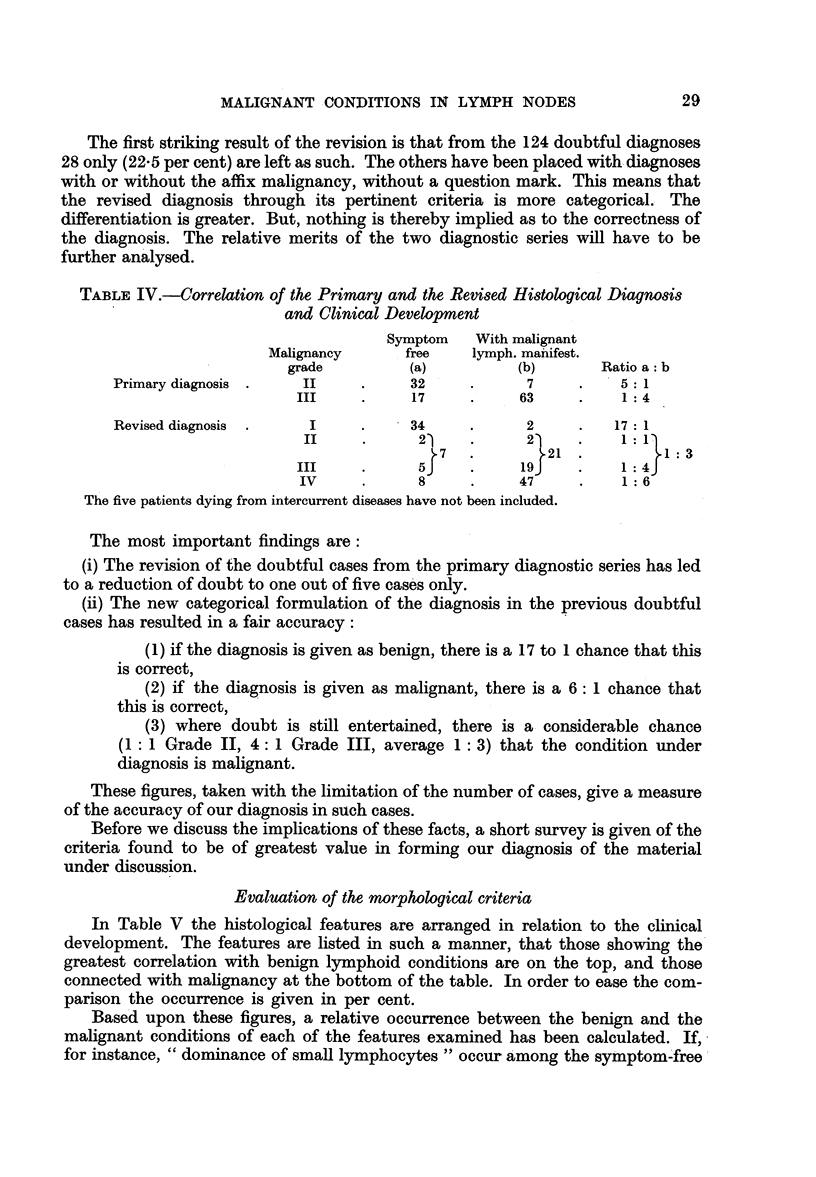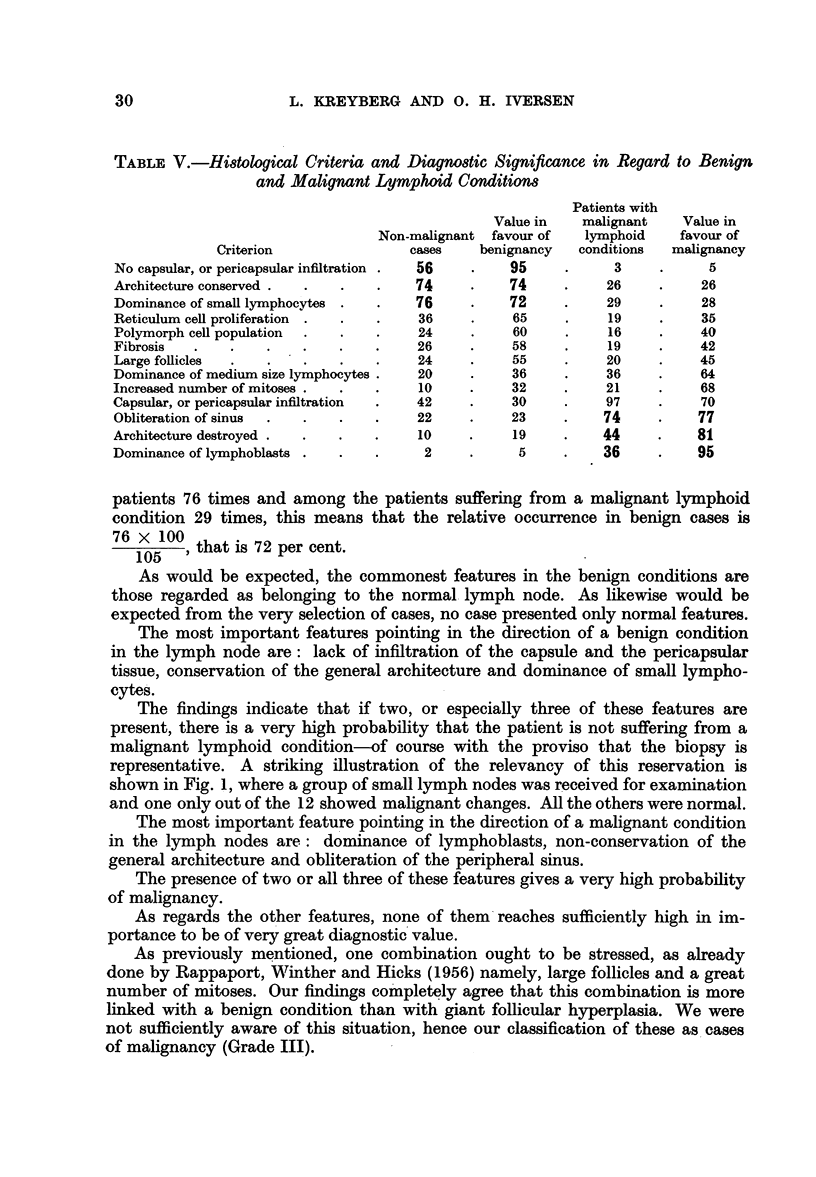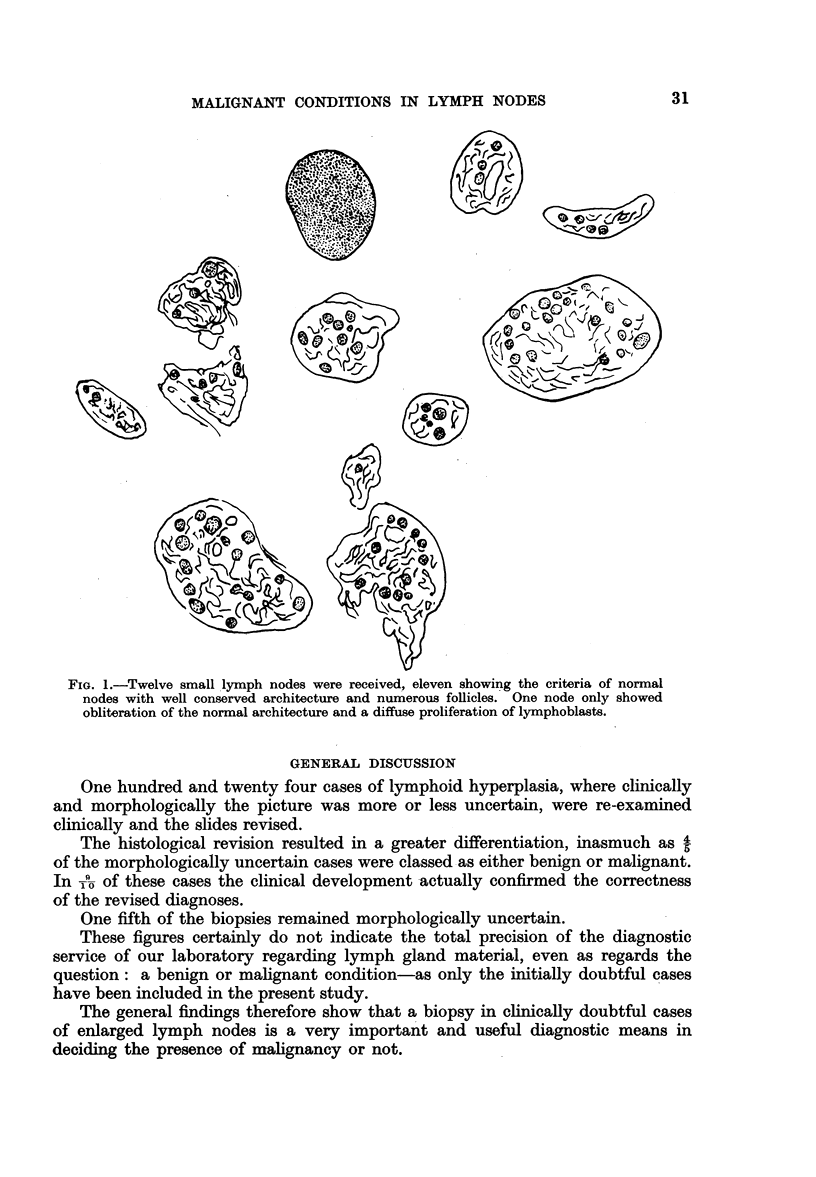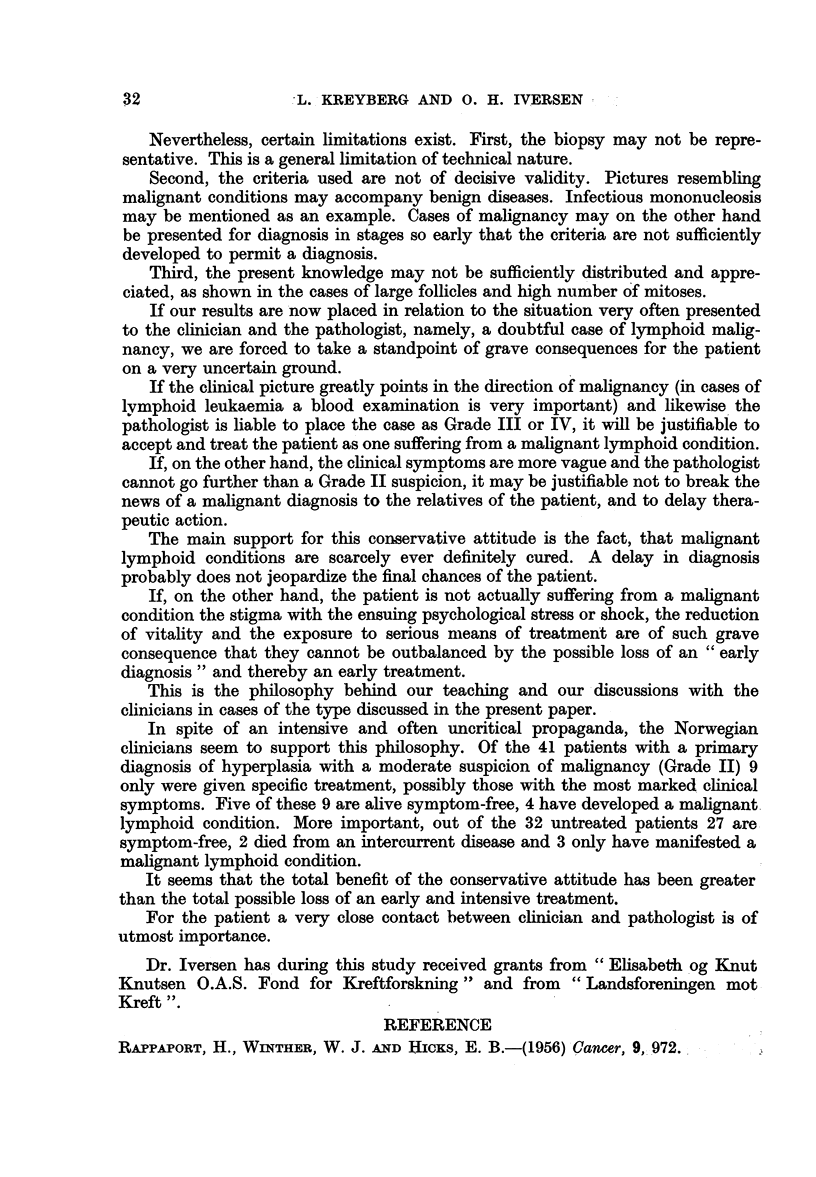# Early Diagnosis of Malignant Conditions in Lymph Nodes

**DOI:** 10.1038/bjc.1959.4

**Published:** 1959-03

**Authors:** L. Kreyberg, O. H. Iversen


### EARLY DIAGNOSIS OF MALIGNANT CONDITIONS IN LYMPH NODES

L. KREYBERG AND O. H. IVERSEN

*From the Institutt for Generell og Eksperimentell Patologi, Universitetet i Oslo*

Received for publication November 1, 1958

The diagnosis of a malignant condition is always of very serious significance. If the diagnosis is correct, the future life of the patient is troubled and the ultimate fate is dark.

But, even if the diagnosis is incorrect, it is accompanied by grave consequences. The patient is stigmatized, he lives in a tense condition full of anxiety, and he is likely to be subject to radical therapeutic steps, to surgery or intensive irradiation, not seldom followed by a more or less marked reduction of his activity and well-being.

In our days the situation is aggravated by the intensive propaganda for " early diagnosis ", as well as by the therapeutic perfectionism aiming at " cure ". The plea for early diagnosis by its own momentum leads to a diagnosis supported by small deviations from the normal, small in quality and small in quantity. The " earlier " the changes, the greater the uncertainty.

This problem often is presented :— which are the therapeutic steps to be taken on the basis of uncertain clinical and histological data ? In few fields is this uncertainty better felt than when lymphoid tissue is involved.

In the present paper an analysis will be made of a series of cases, where the clinician, in the presence of enlarged lymph nodes, decided to have a biopsy examined—and where, after the examination, the pathologist gave a diagnosis of :— " Lymphoid hyperplasia. Malignancy cannot be excluded ".

The material is part of a total of 3483 lymph nodes received at our laboratory in the years 1946–55. Out of this series 124 cases were found, presenting the criteria mentioned.

*The primary diagnosis* in the following is the designation for the original diagnosis, with the accompanying remark. These diagnoses were, during a period of 10 years, given by 5 different pathologists. The diagnostic criteria, the descriptions and the terminology are accordingly not uniform.

The 124 cases can, corresponding to the terms and expressions used, be divided into two grades of uncertainty. Grade II means that the suspicion of malignancy was moderate. Grade III means that the suspicion was grave.

This material was now made the subject of a *revised diagnosis*, performed by one pathologist (K), according to uniform criteria and without any knowledge of the clinical information or of the previous (primary) diagnosis.

Also this material was now " graded ", with Grade I meaning that the morphological criteria does not substantiate a diagnosis of malignancy. Grade IV means an unreserved diagnosis of malignancy. Also this material will have Grades II and III, indicating a slighter or a graver suspicion of malignancy. These Grades II and III are not based upon the same criteria as those of the primary diagnosis, but, it is nevertheless regarded as useful to correlate these grades.

The patients were clinically re-examined and their fates registered. In every case satisfactory information was obtained. Very helpful, as usual, was our Cancer Registry. We are likewise indebted to the clinicians who generously gave us their help.

More than $\frac{2}{3}$ of the cases (86) had an observation period of 5 years, or more, and all cases had an observation period of 3 years or more.

As our study revealed that the patients dying from, or with, a malignant lymphoid disease had an average survival time of 2·57 years and a median survival time of 1·25 years after the biopsy, we regard a limit of 3 years as acceptable.

TABLE I.—*The Result of the Clinical Follow-up*

| Clinical development | Number M. | F. | Total |
|---|---|---|---|
| Symptom-free . . . . . . . . | 29 | 20 | 49 |
| Died from intercurrent disease . . . . | 2 | 3 | 5 |
| Suffering from malignant lymphoid condition . | 10 | 5 | 15 |
| Died from or with malignant lymphoid condition . | 39 | 16 | 55 |

TABLE II.—*The Final Clinical Diagnosis in Cases of an Accepted Malignant Condition*

| | Died | | | Alive | | |
|---|---|---|---|---|---|---|
| | M. | F. | Total | M. | F. | Total |
| Lymphoid leukaemia . . . | 18 | 6 | 24 | 2 | 1 | 3 |
| Lymphosarcoma . . . | 16 | 7 | 23 | 5 | 3 | 8 |
| Giant follicular hyperplasia . | 1 | 0 | 1 | 0 | 0 | 0 |
| Reticulosarcoma . . . | 1 | 2 | 3 | 1 | 0 | 1 |
| Hodgkin's disease . . . | 3 | 1 | 4 | 2 | 1 | 3 |

*Correlation of primary histological diagnosis and final clinical development*

As already mentioned, during the primary diagnostic work, no grading was performed. A grading was, however, done during the present study and based upon the original phrasing of the suspicion of malignancy.

*Grade II*, representing cases with a moderately worded suspicion of malignancy, embraces 41 patients. Two patients died from an intercurrent disease 1 and 11 years after the biopsy was made. Of the remaining 39 patients 7 (18 per cent) developed a malignant lymphoid condition, the others living symptom free.

It may be mentioned that every one of these 7 malignant cases was correctly diagnosed during the revision. This indicates that the histological material actually was representative. The suspicion of malignancy was substantiated in 18 per cent of the cases only.

When 34 patients were under a certain suspicion of malignancy after examination of the morphological material, in spite of the later benign clinical course, this indicates that the diagnosis had been built upon very small deviations from the normal, and that the pathologists involved probably had been influenced either by the case history or by a philosophy of aggravating the diagnosis for "safety sake".

*Grade III*, representing cases with a more strongly worded suspicion of malignancy, embraces 83 patients. Three patients died from intercurrent disease $\frac{1}{2}$, $\frac{1}{2}$ and 1 year after the biopsy. Out of the remaining 80 cases a malignant lymphoid condition was diagnosed in 63, that is in $\frac{4}{5}$ of the cases. Among the 17 symptom free, there are 5 patients with only 3 years of observation, so that a few more may develop malignancy. Two only, of the 17 living symptom free, were given specific treatment. A postulated " cure " can therefore not materially alter the evaluation.

*Correlation of the revised histological diagnosis and the final clinical development*

*Grade I* represents cases where, during revision, the morphological findings did not substantiate a diagnosis of malignancy, a total of 40 cases. Four patients died from intercurrent disease $\frac{1}{2}$, 1, 1, and 11 years after the biopsy. Among the remaining 36 cases 2 only showed development of a malignant lymphoid condition (5 per cent). Both these cases were from the primary diagnosis classified as Grade III.

*Case* 1. A 14 years old boy, with marked clinical symptoms mentioned in the case history submitted to the pathologist. The slide has been restudied several times, but we cannot find a sufficient morphological basis for a diagnosis of malignancy. Most probably the diagnosis was made on non-representative material and a suggestive case history. The lymph nodes had been irradiated.

*Case* 2. A 61 years old man dying of lymphatic leukaemia within one year after the biopsy. The slide has been re-examined several times and is regarded as difficult to interpret owing to a considerable admixture of plasma cells and eosinophiles. In this case a diagnostic failure must be admitted.

*Grade II* embraces only 4 patients, 2 living symptom free, 2 developing a malignant lymphoid condition.

In both cases of malignancy, the lymph nodes were small, the changes minor, but in both cases lymphocytic infiltration of the capsule was present. These two cases should have been referred to Grade III.

*Grade III* embraces 24 patients out of which 19 ($\frac{4}{5}$) showed a malignant lymphoid condition, whereas 5 patients remained symptom free in the period of observation 10, 8, 7, 5 and 3 years after the biopsy.

In the slides from these patients four cases gave the picture of a well conserved architecture of the lymph nodes, but the follicles were considerably enlarged and showed a great number of mitoses. This finding will be commented upon later.

In the fifth case the histological picture was very like that of the case in Grade II where leukaemia developed later. Here again the criteria did not permit a correct diagnosis.

*Grade IV*, representing cases where the malignancy diagnosis was now given unreservedly, embraces 56 cases, with one patient dying from an intercurrent disease $\frac{1}{2}$ year after the biopsy. Of the 55 remaining, 47 showed a malignant lymphoid condition, whereas 8 remained symptom free. One of the latter suffered from infectious mononucleosis, which means a definite complicating factor for a correct diagnosis. In the other cases the histological picture was still after several revisions regarded as being of a grave character. The observation periods were one case 11 years, two cases 8 years, one case 7 years, one case 4 years, and two cases 3 years.

TABLE III.—*Results of Histological Grading*

| Malignancy grade | Series: Primary | | Series: Revised | |
|---|---|---|---|---|
| I | — | | 40 | |
| II | 41 | } 124 | 4 | } 28 |
| III | 83 | | 24 | |
| IV | — | | 56 | |

The first striking result of the revision is that from the 124 doubtful diagnoses 28 only (22·5 per cent) are left as such. The others have been placed with diagnoses with or without the affix malignancy, without a question mark. This means that the revised diagnosis through its pertinent criteria is more categorical. The differentiation is greater. But, nothing is thereby implied as to the correctness of the diagnosis. The relative merits of the two diagnostic series will have to be further analysed.

TABLE IV.—*Correlation of the Primary and the Revised Histological Diagnosis and Clinical Development*

| | Malignancy grade | Symptom free (a) | With malignant lymph. manifest. (b) | Ratio a : b |
|---|---|---|---|---|
| Primary diagnosis | II | 32 | 7 | 5 : 1 |
| | III | 17 | 63 | 1 : 4 |
| Revised diagnosis | I | 34 | 2 | 17 : 1 |
| | II | 2 | 2 | 1 : 1 |
| | (II + III) | } 7 | } 21 | } 1 : 3 |
| | III | 5 | 19 | 1 : 4 |
| | IV | 8 | 47 | 1 : 6 |

The five patients dying from intercurrent diseases have not been included.

The most important findings are :

(i) The revision of the doubtful cases from the primary diagnostic series has led to a reduction of doubt to one out of five cases only.

(ii) The new categorical formulation of the diagnosis in the previous doubtful cases has resulted in a fair accuracy :

(1) if the diagnosis is given as benign, there is a 17 to 1 chance that this is correct,

(2) if the diagnosis is given as malignant, there is a 6 : 1 chance that this is correct,

(3) where doubt is still entertained, there is a considerable chance (1 : 1 Grade II, 4 : 1 Grade III, average 1 : 3) that the condition under diagnosis is malignant.

These figures, taken with the limitation of the number of cases, give a measure of the accuracy of our diagnosis in such cases.

Before we discuss the implications of these facts, a short survey is given of the criteria found to be of greatest value in forming our diagnosis of the material under discussion.

*Evaluation of the morphological criteria*

In Table V the histological features are arranged in relation to the clinical development. The features are listed in such a manner, that those showing the greatest correlation with benign lymphoid conditions are on the top, and those connected with malignancy at the bottom of the table. In order to ease the comparison the occurrence is given in per cent.

Based upon these figures, a relative occurrence between the benign and the malignant conditions of each of the features examined has been calculated. If, for instance, " dominance of small lymphocytes " occur among the symptom-free

TABLE V.—*Histological Criteria and Diagnostic Significance in Regard to Benign and Malignant Lymphoid Conditions*

| Criterion | Non-malignant cases | Value in favour of benignancy | Patients with malignant lymphoid conditions | Value in favour of malignancy |
|---|---|---|---|---|
| No capsular, or pericapsular infiltration | **56** | **95** | 3 | 5 |
| Architecture conserved | **74** | **74** | 26 | 26 |
| Dominance of small lymphocytes | **76** | **72** | 29 | 28 |
| Reticulum cell proliferation | 36 | 65 | 19 | 35 |
| Polymorph cell population | 24 | 60 | 16 | 40 |
| Fibrosis | 26 | 58 | 19 | 42 |
| Large follicles | 24 | 55 | 20 | 45 |
| Dominance of medium size lymphocytes | 20 | 36 | 36 | 64 |
| Increased number of mitoses | 10 | 32 | 21 | 68 |
| Capsular, or pericapsular infiltration | 42 | 30 | 97 | 70 |
| Obliteration of sinus | 22 | 23 | **74** | **77** |
| Architecture destroyed | 10 | 19 | **44** | **81** |
| Dominance of lymphoblasts | 2 | 5 | **36** | **95** |

patients 76 times and among the patients suffering from a malignant lymphoid condition 29 times, this means that the relative occurrence in benign cases is $\frac{76 \times 100}{105}$, that is 72 per cent.

As would be expected, the commonest features in the benign conditions are those regarded as belonging to the normal lymph node. As likewise would be expected from the very selection of cases, no case presented only normal features.

The most important features pointing in the direction of a benign condition in the lymph node are: lack of infiltration of the capsule and the pericapsular tissue, conservation of the general architecture and dominance of small lymphocytes.

The findings indicate that if two, or especially three of these features are present, there is a very high probability that the patient is not suffering from a malignant lymphoid condition—of course with the proviso that the biopsy is representative. A striking illustration of the relevancy of this reservation is shown in Fig. 1, where a group of small lymph nodes was received for examination and one only out of the 12 showed malignant changes. All the others were normal.

The most important feature pointing in the direction of a malignant condition in the lymph nodes are: dominance of lymphoblasts, non-conservation of the general architecture and obliteration of the peripheral sinus.

The presence of two or all three of these features gives a very high probability of malignancy.

As regards the other features, none of them reaches sufficiently high in importance to be of very great diagnostic value.

As previously mentioned, one combination ought to be stressed, as already done by Rappaport, Winther and Hicks (1956) namely, large follicles and a great number of mitoses. Our findings completely agree that this combination is more linked with a benign condition than with giant follicular hyperplasia. We were not sufficiently aware of this situation, hence our classification of these as cases of malignancy (Grade III).

FIG. 1.—Twelve small lymph nodes were received, eleven showing the criteria of normal nodes with well conserved architecture and numerous follicles. One node only showed obliteration of the normal architecture and a diffuse proliferation of lymphoblasts.

### GENERAL DISCUSSION

One hundred and twenty four cases of lymphoid hyperplasia, where clinically and morphologically the picture was more or less uncertain, were re-examined clinically and the slides revised.

The histological revision resulted in a greater differentiation, inasmuch as $\frac{4}{5}$ of the morphologically uncertain cases were classed as either benign or malignant. In $\frac{9}{10}$ of these cases the clinical development actually confirmed the correctness of the revised diagnoses.

One fifth of the biopsies remained morphologically uncertain.

These figures certainly do not indicate the total precision of the diagnostic service of our laboratory regarding lymph gland material, even as regards the question : a benign or malignant condition—as only the initially doubtful cases have been included in the present study.

The general findings therefore show that a biopsy in clinically doubtful cases of enlarged lymph nodes is a very important and useful diagnostic means in deciding the presence of malignancy or not.

Nevertheless, certain limitations exist. First, the biopsy may not be representative. This is a general limitation of technical nature.

Second, the criteria used are not of decisive validity. Pictures resembling malignant conditions may accompany benign diseases. Infectious mononucleosis may be mentioned as an example. Cases of malignancy may on the other hand be presented for diagnosis in stages so early that the criteria are not sufficiently developed to permit a diagnosis.

Third, the present knowledge may not be sufficiently distributed and appreciated, as shown in the cases of large follicles and high number of mitoses.

If our results are now placed in relation to the situation very often presented to the clinician and the pathologist, namely, a doubtful case of lymphoid malignancy, we are forced to take a standpoint of grave consequences for the patient on a very uncertain ground.

If the clinical picture greatly points in the direction of malignancy (in cases of lymphoid leukaemia a blood examination is very important) and likewise the pathologist is liable to place the case as Grade III or IV, it will be justifiable to accept and treat the patient as one suffering from a malignant lymphoid condition.

If, on the other hand, the clinical symptoms are more vague and the pathologist cannot go further than a Grade II suspicion, it may be justifiable not to break the news of a malignant diagnosis to the relatives of the patient, and to delay therapeutic action.

The main support for this conservative attitude is the fact, that malignant lymphoid conditions are scarcely ever definitely cured. A delay in diagnosis probably does not jeopardize the final chances of the patient.

If, on the other hand, the patient is not actually suffering from a malignant condition the stigma with the ensuing psychological stress or shock, the reduction of vitality and the exposure to serious means of treatment are of such grave consequence that they cannot be outbalanced by the possible loss of an " early diagnosis " and thereby an early treatment.

This is the philosophy behind our teaching and our discussions with the clinicians in cases of the type discussed in the present paper.

In spite of an intensive and often uncritical propaganda, the Norwegian clinicians seem to support this philosophy. Of the 41 patients with a primary diagnosis of hyperplasia with a moderate suspicion of malignancy (Grade II) 9 only were given specific treatment, possibly those with the most marked clinical symptoms. Five of these 9 are alive symptom-free, 4 have developed a malignant lymphoid condition. More important, out of the 32 untreated patients 27 are symptom-free, 2 died from an intercurrent disease and 3 only have manifested a malignant lymphoid condition.

It seems that the total benefit of the conservative attitude has been greater than the total possible loss of an early and intensive treatment.

For the patient a very close contact between clinician and pathologist is of utmost importance.

Dr. Iversen has during this study received grants from " Elisabeth og Knut Knutsen O.A.S. Fond for Kreftforskning " and from " Landsforeningen mot Kreft ".

### REFERENCE

Rappaport, H., Winther, W. J. and Hicks, E. B.—(1956) *Cancer*, **9**, 972.